# Pathogenicity and virulence of malaria: Sticky problems and tricky solutions

**DOI:** 10.1080/21505594.2022.2150456

**Published:** 2023-01-04

**Authors:** Isobel S Walker, Stephen J Rogerson

**Affiliations:** Department of Infectious Diseases, The University of Melbourne, The Doherty Institute, Melbourne, Australia

**Keywords:** *Plasmodium falciparum*, *Plasmodium vivax*, severe malaria, *Plasmodium falciparum* erythrocyte membrane protein 1, var genes, sequestration, cytokine, hypnozoite

## Abstract

Infections with *Plasmodium falciparum* and *Plasmodium vivax* cause over 600,000 deaths each year, concentrated in Africa and in young children, but much of the world’s population remain at risk of infection. In this article, we review the latest developments in the immunogenicity and pathogenesis of malaria, with a particular focus on *P. falciparum*, the leading malaria killer. Pathogenic factors include parasite-derived toxins and variant surface antigens on infected erythrocytes that mediate sequestration in the deep vasculature. Host response to parasite toxins and to variant antigens is an important determinant of disease severity. Understanding how parasites sequester, and how antibody to variant antigens could prevent sequestration, may lead to new approaches to treat and prevent disease. Difficulties in malaria diagnosis, drug resistance, and specific challenges of treating *P. vivax* pose challenges to malaria elimination, but vaccines and other preventive strategies may offer improved disease control.

## Introduction

There are 241 million cases of malaria per year, predominantly in sub-Saharan Africa. Malaria can be life threatening and caused 627,000 deaths in 2021 [1]. Of the five species of *Plasmodium spp*. that commonly infect humans – *P. falciparum, P. vivax, P. ovale, P. malariae, and P. knowlesi* – *P. falciparum* causes 95% of the cases and the great majority of the severe disease [1], although severe disease can also be caused by the less prevalent *P. vivax* and *P. knowlesi* [2,3].

Malaria is one of the oldest diseases on record and has shaped the human genome, leading to the selection of protective genetic traits such as sickle cell trait and beta-thalassaemia. Similarly, *Plasmodium spp*. have developed mechanisms to prolong infection in the human host, including the ability to evade immunity by expressing highly diverse surface antigens and to sequester in the host microvasculature, and continue to develop resistance to chemotherapeutics. This review will summarize the key virulence factors involved in progression to or protection from severe disease and highlight important areas of future research.

## Epidemiological risk factors for severe disease

Clinical manifestations of malaria range from mild to life threatening. Uncomplicated malaria symptoms are non-specific and include fever, nausea, vomiting, headache, and diarrhoea, with no indication of organ dysfunction. Uncomplicated malaria can rapidly become severe malaria, defined by the World Health Organization (WHO) as one or more of a range of conditions, including impaired consciousness, respiratory distress, convulsions, prostration, shock, pulmonary oedema, abnormal bleeding, and jaundice, that can be recognized by several laboratory indicators including severe anaemia, hyperparasitaemia, hyperlactaemia, acidosis, and renal impairment [4].

The incidence of severe malaria is higher in high transmission settings [5] and children under 5 are most susceptible [6]. As such, children under 5 account for approximately 40% of global malaria cases and the majority of malaria related deaths [1]. In high transmission settings, *Plasmodium spp*. infections in adults are generally asymptomatic, due to acquisition of protective immunity after repeat infections. In areas of low or unstable transmission, or in previously unexposed individuals, older children and adults are also vulnerable to severe disease [5,7]. In addition to young children, pregnant women are highly susceptible to *P. falciparum* infection and severe *P. falciparum* malaria, particularly severe anaemia. Malaria during pregnancy can also affect the baby, by causing miscarriage, stillbirth, or low birthweight delivery, which predisposes to neonatal and infant mortality [8].

There are many environmental factors that contribute to transmission of malaria, such as rainfall, proximity to stagnant water, and human activity. In general, urbanization is thought to be a key contributor to the reduction of malaria [9] and urban areas have less transmission compared to rural areas [10]. However, some city centres have high estimated inoculation rates, likely due to several factors including poor drainage, unplanned urban dwellings [10,11] or nearby deforestation [12]. Additionally, *Anopheles* mosquitoes have adapted to breed in urban environments, such as polluted water and insecticide treated water [13,14]. *Anopheles stephensi* is a prevalent vector for both *P. falciparum and P. vivax* malaria in South Asia that is well adapted to breed in urban settings [15] and has expanded to the Horn of Africa where it is predicted to continue to spread and increase urban malaria outbreaks [16].

## Diagnostics

Prompt treatment is key to averting severe malaria and relies on access to accurate diagnosis and effective therapeutics. Blood-stage malaria can be diagnosed by light microscopic examination of stained thick and thin smears of peripheral blood, and microscopy remains the field standard for malaria diagnostics. However, light microscopy requires electricity and false negatives and incorrect speciation are common in microscopic diagnosis of *P. malariae*, *P. ovale,* and *P. vivax*, due to low density and similarities in morphology [17]. Microscopy is also unlikely to detect low-density “sub-microscopic” infections (approximately <100 parasites per microliter of blood) that are reservoirs for transmission and are important to identify in countries targeting malaria elimination; in screening pregnant women who may have parasite sequestered in the placenta; as well as for blood safety in transfusion [18]. Ultrasensitive PCR offers higher sensitivity than microscopy but the reliance on electricity, costly reagents and laboratory facilities for sample preparation have limited PCR to reference laboratories, rather than point of care [19]. Loop-mediated isothermal amplification (LAMP) is a more portable alternative to PCR but the high cost and sample preparation requirements have prevented extensive use in the field, although a recent study suggests it may be cost effective to include in surveillance programs [20].

Rapid diagnostic tests (RDTs) are affordable and easy to use and a large number of RDTs are commercially available that meet the WHO minimum performance criteria [21]. Malaria RDTs mostly detect *P. falciparum* histidine-rich protein 2 (HRP2), as well as *P. falciparum* lactate dehydrogenase (LDH), *P. vivax* LDH, pan species LDH, and aldolase [21]. Similarly to microscopy, RDTs lack sensitivity to detect low-density infections [18], limiting their use in elimination settings. They are further challenged by the presence of HRP2 and/or HRP3 deletions in South America and sub-Saharan Africa [22]. HRP2 antibodies can cross react with HRP3 at a certain antigen threshold and so RDTs can still be effective in regions with only HPR2 or HRP3 deletions, but not both [23]. Interestingly, both modelling and field studies have shown that HRP2 deletions are driven by low transmission and the use of PfHRP2 RDTs to correctly treat a high proportion of infections [24–26].

## Life cycle and parasite density

### Life cycle

Malaria was first confirmed to be caused by the *Plasmodium* parasite when observed by microscopy in a peripheral blood smear, in 1880 [27]. Female *Anopheles* mosquitoes transmit the *sporozoite* form of *P. falciparum* to humans with saliva whilst taking a blood feed. Sporozoites are activated by migrating through hepatocytes and Kupffer cells [28]. The activated sporozoites replicate by mitosis in hepatocytes, producing up to 40,000 parasites per hepatocyte, for 1–2 weeks. Hepatocytes release merozoite filled merosomes into the blood stream where they infect and replicate within erythrocytes. *P. vivax* and *P. ovale* can remain dormant (non-replicating) in the liver as hypnozoites for weeks, months and sometimes years following infection and can reactivate to cause relapses, although the mechanisms by which this occurs are unclear.

Malaria symptoms occur during the blood stage of the parasite lifecycle, a process which repeatedly cycles every 24–72 h, depending on the species. *P. falciparum* merozoites invade erythrocytes of all ages, whereas *P. vivax* will only invade reticulocytes recently released from the bone marrow. Merozoites use an actomyosin motor to undergo gliding motility across erythrocyte surfaces before attachment and invasion [29]. Invasion is a multi-step process involving complex protein interactions. Initial low affinity attachment to the erythrocyte surface is followed by reorientation to align the apical end of the merozoite with the erythrocyte surface; a “tight junction” of high affinity interactions is then formed and finally, the merozoite surface is “shed” as it enters the erythrocyte [30]. During the first hours inside the erythrocyte, the parasite is recognizable by a ring shape under Giemsa staining and is known as the *ring* stage. Ring-stage parasites establish a protein trafficking network and begin remodelling the infected erythrocyte (IE) [31].

In *P. falciparum* infection, as rings develop into *trophozoite* stage parasites, they begin exporting knob-associated histidine-rich protein through the red cell cytoplasm to the plasma membrane which becomes deformed with knob-like protrusions (knobs) [32]. Knobs are unique to *P. falciparum*, and anchor *P. falciparum* erythrocyte membrane protein 1 (PfEMP1) – a key parasite virulence factor – on the IE surface during the *trophozoite* stage [33]. Trophozoites develop into *schizonts*, which burst to release 8–32 merozoites that infect erythrocytes and continue the asexual lifecycle. The parasite metabolizes host haemoglobin during the blood stage and detoxifies the released free haem by polymerization into a pigmented crystal known as haemozoin [34].

A small percentage of ring-stage parasites differentiate into male or female *gametocytes* (reviewed in [35]), the sexual form of the parasite, driven in part by the parasite’s ability to sense host physiological signals [36]. Gametocytes are taken up by mosquitoes during a blood meal and enter the mosquito midgut. Gametocytes shed the erythrocyte membrane and the released male *microgametes* fertilize the released *macrogametes* to form a *zygote*, which develops into a motile *ookinete*. *Ookinetes* invade the midgut wall and become *oocysts*, which mitotically replicate and eventually rupture to release sporozoites. Sporozoites migrate to, and remain in, the salivary glands to be transmitted to a human host [37].

### Parasite biomass

**Figure 1.**
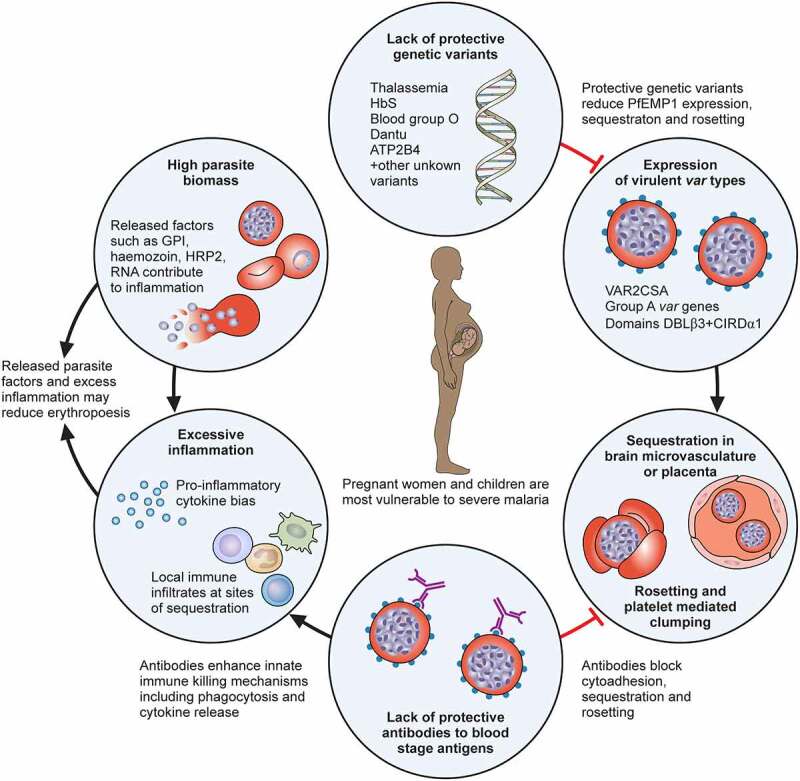
Key pathogenic factors that contribute to severe malaria. High parasite biomass and parasite products released into circulation during cell lysis are stimulants for inflammation and may directly contribute to severe anaemia by reducing erythropoiesis. A strong inflammatory response, including Th1 type cytokine bias and sequestration of innate immune cells, such as monocytes, can activate and damage endothelial cells, enhance sequestration and contribute to severe anaemia by excessive complement mediated lysis of uninfected erythrocytes. A lack of antibodies targeting IE surface antigens may contribute to high parasite biomass and sequestration, although the key targets of antibodies are unclear. Sequestration of IE, mediated by PfEMP1, allows the parasite to evade clearance by the spleen and sequestration in the brain of young children is key to the pathogenesis of cerebral malaria. Sequestration in the placenta is strongly associated with poor pregnancy outcomes. Sequestration in the placenta is mediated by PfEMP1 expressed from *VAR2CSA* and sequestration in the brain microvasculature is associated with Group A PfEMP1 with domains that bind to ICAM-1 and EPCR. Protective genetic variants can reduce PfEMP1 expression, sequestration and rosetting.

It has long been established that a high parasite load is associated with poorer clinical outcomes, and hyperparasitaemia (>10% parasitaemia) is one of the WHO criteria for severe malaria (Figure 1) [4]. Circulating parasitaemia does not accurately reflect total parasites in the body as *P. falciparum* sequesters in the microvasculature, out of circulation. Instead, plasma level of HRP2 is a stronger correlate of severe malaria and predictor of mortality than circulating parasitaemia [38,39]. The relationship between HRP2 and severity of malaria has been reported in several studies [39–41], although others have found no relationship [42]. A similar relationship has also been observed for *P. vivax*, in which circulating *P. vivax* specific LDH reflects the emerging importance of a sequestered parasite biomass in the spleen and bone marrow (as will be discussed below) [43].

The threshold at which parasitaemia is associated with fever increases with age and is higher in holoendemic settings than mesoendemic settings [44], suggesting that exposure-dependent acquisition of immunity is a key regulator or parasite density. Other factors including parasite multiplication rate, sequestration of mature IEs, and RBC mutations likely also contribute to parasitaemia, as will be discussed in this review.

The clinical impact of low-density infections is unclear. In a study of 2801 febrile children, there were no differences in severe clinical outcomes or secondary hospitalizations for individuals with low-density *P. falciparum (*who were not treated with antimalarials) compared to uninfected children [45]. In a 2-year longitudinal study, persistent, low-density infections were found to oscillate between low and high density over time but individuals with oscillating parasitaemia remained afebrile and were untreated [46]. In contrast, submicroscopic infection in Ugandan children was associated with both febrile and non-febrile illness [47]. The consequences or benefits of low-density or chronic asymptomatic infection are largely unknown, although some researchers believe that all such infections should be treated [48].

In the absence of mass drug administration regimes, low-density infections are untreated. Although individuals with submicroscopic infection are less likely to infect mosquitoes than microscopy detected infection [49], submicroscopic infections can carry gametocytes [50] and therefore are a potential “reservoir” for transmission. In a low, seasonal transmission area of Vietnam, 9% of *P. falciparum* and 46% of *P. vivax* infections lasted for over 6 months (during periods of low transmission) [46], suggesting that chronic, low-density infections may maintain endemicity between malaria seasons.

## Genetic variation in red blood cells

Several RBC polymorphisms occur at higher frequency amongst populations that have historically experienced a high burden of malaria and therefore are likely to offer an evolutionary survival advantage [51]. It is well established that thalassaemia (reduced α or β haemoglobin) and heterozygous sickle cell haemoglobin (HbS) confer significant protection from severe malaria and are widespread in historically malaria endemic settings, particularly the Mediterranean and Sub Saharan Africa [52]. Blood group O is also more common amongst malaria endemic populations and has been associated with protection from severe malaria [51]. Interestingly, a recent study found a strong positive correlation between children with HbS and 3 genetic variants in the parasite genome and the protective effect of HbS against severe malaria was not observed in the presence of these parasite variants. This small study suggests a more complex evolutionary competition between host and parasite genomes may be at play [53].

There are likely to be a number of host genetic traits that contribute to protection and are not yet characterized [51]. Of recent interest, populations with Dantu (a polymorphism in RBC surface glycophorins) and mutations in the RBC membrane protein ATP2B4 have been associated with protection from severe malaria [54–56]. However, these and other known polymorphisms (including HbS) only account for 5% of the total estimated genetic effect on the risk of severe malaria [54], suggesting there are many other genetic variants influencing malaria pathogenesis.

Understanding the mechanisms by which naturally selected genetic traits contribute to protection enhances our understanding of malaria pathogenesis that may open doors to novel therapeutic strategies. For example, the Dantu mutation increases membrane tension and in turn inhibits merozoite invasion, suggesting that a membrane tension threshold may be a key factor for successful merozoite invasion [57]. Several mechanisms of protection for haemoglobin mutations have been proposed, including reduced haemoglobin digestion by parasites and increased splenic clearance [51]. Studies have shown that sickle cell trait [58], haemoglobin C [59] and a gain-of-function mutation in the mechanosensitive ion channel *PIEZO1 E756del* [60] reduce surface presentation of the major variant surface antigen PfEMP1, in turn reducing adhesion to host receptors involved in parasite sequestration [61]. In the case of blood types, IE have impaired ability to form rosettes (the adhesion of IE to uninfected erythrocytes) in blood group O erythrocytes [62]. Both rosetting and sequestration are important virulence mechanisms that will be discussed below.

## Cytoadherence, clumping, and rosetting

### Cytoadherence and sequestration

Asexual *P. falciparum* IEs sequester in the heart, lungs, brain, skin, gastrointestinal tract, placenta, and other organs [63]. Sequestration is generally thought of as a mechanism to avoid clearance by the spleen and may help avoid detection by circulating immune cells [64]. Some sequestration sites have been linked to severe clinical outcomes, including cerebral malaria and placental malaria (as will be discussed in section 7).

Sequestration occurs when *P. falciparum* IE cytoadhere to receptors on endothelial cells or syncytiotrophoblast lining the placenta via cell surface antigens, particularly PfEMP1. Several possible endothelial adhesion receptors have been identified by *in vitro* and post-mortem histological studies, including Cluster of Differentiation 36 (CD36), intercellular adhesion molecule-1 (ICAM-1), E selectin, P-Selectin, platelet endothelial cell adhesion molecule-1 (PECAM-1), vascular cell adhesion molecule-1 (VCAM-1), endothelial protein C receptor (EPCR), integrins and fibronectins [65], while chondroitin sulphate A (CSA) mediates placental sequestration [33]. In addition to PfEMP1-mediated adhesion, the altered structure of the IE compared to non-IE is also important for sequestration. Knobless parasites that express PfEMP1 have impaired infectivity in monkeys [66] and in humans [67,68] and this is thought to be due to reduced sequestration, allowing for efficient splenic clearance. Using a 3D in vitro model of the arteriole-capillary venules, parasites lacking knobs or PfEMP1 had reduced sequestration in narrow capillary constriction zones [69]. The authors suggest a mechanism whereby reduced deformability and irregular surface structure decelerates IE flow in capillaries to allow for PfEMP1 adhesion and sequestration in post capillary venules [69].

In addition to asexual parasites, post-mortem histology has revealed sequestration of immature gametocyte IE in the extravascular bone marrow parenchyma [70], accounting for the lack of immature gametocytes seen in circulation. This has led to the hypothesis that gametocytes mature in the bone marrow parenchyma and re-enter circulation in preparation for being taken up by the mosquito. The mechanisms of sequestration of sexual stage parasites are unclear. In mice, *P. berghei* migrate through the sinusoidal barrier of the bone marrow, spleen, and liver and translocation into the bone marrow parenchyma was partially dependent on host P-selectin expression [71]. Adhesion of gametocytes to human endothelial cell lines has been observed in some studies [72] but not others [73]. A recent study found that immature but not mature gametocytes bind to primary human bone marrow mesenchymal stromal cells *in vitro* and this activated the release of cytokines and growth factors, many of which are involved in angiogenesis [74]. Adhesion was mediated by a trypsin-sensitive surface molecule that is unlikely to be PfEMP1, due to its low expression on gametocyte IEs [74]. Gametocyte sequestration in bone marrow may be critical to sexual development and a clearer understanding of the mechanisms of sequestration may lead to opportunities for transmission blocking therapies. Interestingly, a recent study found that primaquine reacts with cytochrome P450 2D6 (CYP2D6), followed by cytochrome P450 NADPH:oxidoreductase (CPR) to generate excessive H_2_O_2_ that ultimately kills gametocytes, and proposed that the colocalization of CYP2D6 and CPR in the liver and bone marrow explains the efficacy of primaquine to kill hypnozoites and *P. falciparum* and *P. vivax* gametocytes [75].

### Sequestration in *P. vivax*

Asexual *P. vivax-*infected reticulocytes do not display PfEMP1-like antigens or knobs; however, there is some evidence for their cytoadherence to lung and placental endothelial cells *in vitro* [76]. Low levels of apparent sequestration of asexual parasites have been observed *ex vivo* in the lung [77], liver, and bone marrow parenchyma [78], and like *P. falciparum*, immature *P. vivax* gametocytes sequester in the bone marrow parenchyma as a potential site of development and reservoir for infection [78,79].

Interestingly, a recent study of *Plasmodium spp*. infected, asymptomatic individuals undergoing splenectomy found massive sequestration of intact *P. vivax* IE of all stages in the spleen, at a rate 3590 times higher than the peripheral blood [80]. *P. falciparum* IE also sequestered in the spleen but to a far lesser extent [80]. It remains open to speculation whether these parasites can enter circulation and how this influences disease outcome.

### Sequestration and the spleen

The spleen has multiple important functions in infection. Splenic macrophages and polymorphonuclear cells remove IE by phagocytosis [81] and this may be impaired at high parasitaemia [82]. The spleen also recycles erythrocytes by “pitting”- squeezing the IE through narrow passages into the red pulp sinuses that removes trophozoite, schizont, and some ring-stage parasites and releases the uninfected cell portion into circulation [83,84]. Studies of splenectomies in humans and monkeys have found that the spleen also modulates expression of knobs and variant surface antigens in *P. falciparum* IE [82,85] and modulates expression of parasite factors in *P. vivax* [86]. This suggests that although the spleen may help the host by clearing or possibly with-holding parasites from circulation to minimize sequestration at other sites, sequestration in the spleen may perpetuate infection by acting as a chronic reservoir or by increasing surface antigen and knob expression that leads to sequestration.

### Rosetting and clumping

Trophozoite IE infected with *P. falciparum* can bind to two or more uninfected red blood cells to form “rosettes.” This phenomenon is observed with some parasite lines but not others, suggesting it is a variant specific mechanism. Rosetting is also not associated with parasite density [87–89], suggesting rosetting specific epitopes are involved rather than non-specific aggregation due to morphological or other generic changes. Rosetting is probably mediated by both variants of PfEMP1 and RIFINS (repetitive interspersed family proteins), although STEVORs (subtelomeric variable open reading frame proteins) may also be involved [62,90]. Rosetting is enhanced in non-blood group O compared to blood group O erythrocytes [62], in particular blood group A and AB increase rosette rate and rosette strength [91,92]. Rosettes still form in blood group O suggesting other RBC surface receptors are also involved, and complement receptor 1, glycosaminoglycans (heparan sulphate) and glycophorin C on the surface of uninfected erythrocytes have been implicated [62]. Serum factors including IgM and α_2_ macroglobulin binding to IEs can also mediate adhesion to uninfected erythrocytes in laboratory adapted and field isolates [93,94].

In several studies, rosetting was more common in isolates taken from individuals with cerebral malaria or with severe malaria anaemia [87–89] but not in others [95]. In line with the importance of blood group antigens and rosetting in severe malaria, blood group A/B/AB are at greater risk of severe malaria compared to blood group O [96].

The mechanisms by which rosetting contributes to severe malaria are still unclear. Rosetting has been hypothesized to block or slow capillary blood flow, possibly enhancing cytoadherence [97]. Rosetting may also inhibit immune mechanisms of parasite clearance. Non-O blood group rosettes block parasite epitopes (PfEMP1) from antibody binding and heparin disruption [92,98] and rosetting has been shown to reduce phagocytosis of IEs [99,100]. In a recent study, artesunate stimulated rosetting and artemisinin resistant parasites formed rosettes faster and at a higher rate than non-resistant parasites. The authors propose that rapid and frequent rosetting protects resistant parasites from intracellular artemisinin accumulation to perpetuate resistance [100].

Rosetting has been occasionally described in other parasite species [101], although its importance is less well understood. Rosettes produced by clinical isolates of *P. vivax* were similar in strength to those of *P. falciparum* and may alter IE deformability to enhance sequestration [102]. Rosetting may be mediated by glycophorin C receptor or serum factors such as IgM [62,99]. *P. vivax* rosetting has been associated with anaemia [103]; however, the contribution to severe disease, sequestration in the spleen or bone marrow, or influence on parasite load *in vivo* requires further study.

Aside from adhesion to uninfected erythrocytes, parasite IEs are also able to bind to CD36 or gC1qR/HABP1 on the surface of platelets to mediate clumping of IEs [104,105]. Clumping of IEs has been shown to be more common in parasites causing severe malaria than those causing uncomplicated malaria in some studies [104,106]. However, clumping was shown to be strongly associated with parasitaemia, rather than disease outcome, in another study [107]. High assay variability *in vitro* makes it difficult to discern the importance of clumping in severe malaria [108], and it is unknown whether specific PfEMP1 or other IE surface receptors are involved.

## Virulent surface antigen diversity

Variant surface antigens (VSAs) are central to the pathogenesis of *P. falciparum* malaria. There are 4 main families of VSAs expressed during the blood stage of *P. falciparum*: PfEMP1, encoded by   60 *var* genes; RIFINs, encoded by 150–200 *rif* genes; Surface-associated interspersed gene family proteins (SURFINs), encoded by   10 *surf* genes; and STEVOR, encoded by 30–40 *stevor* genes [109]. The large variation in surface antigens gives the parasite a survival advantage by evading epitope recognition and destruction by the humoral immune system [110,111]. Bull et al. showed that at the start of an infection, children had significantly fewer agglutinating antibodies to the IEs causing their infection than to other parasite isolates from the community, supporting the idea that the parasite exploits “holes in the antibody repertoire” [112]. PfEMP1 is generally considered the most important VSA family due to its cytoadhering properties. In general, only one PfEMP1 type is expressed by a single parasite and there is often a dominant transcript in an infected individual. In addition to switching of PfEMP1 expression upon a5ntibody recognition, PfEMP1 expression is influenced by availability of adhesion receptors. I*n vitro*, cultures of IEs panned on a particular receptor will favour expression of PfEMP1 that can adhere to the receptor [113]. A well-characterized example *in vivo* is that *var2csa* becomes the dominant *var* gene to be expressed by parasites infecting pregnant women and encodes for PfEMP1 that binds to placental CSA receptors [114]. The wide variation in *var* genes allows the parasite to adapt to different adhesion environments to avoid splenic clearance. Additionally, multiple *var* genes of the same binding phenotype exist, allowing the parasite to maintain adhesion to particular receptors despite antibody recognition [115].

Multigene families have been identified in *P. vivax*, including the *vir* gene family, but are less well characterized due to high antigenic variation and difficulty maintaining cultures *in vitro*. Different VIR protein subfamilies are trafficked to different cellular locations, including the reticulocyte surface (for VIR14 of subfamily C) where they have been speculated to be involved in cytoadherence [116]. Transgenic *P. falciparum* IE expressing VIR14 has been shown to bind to ICAM-1 and human spleen fibroblasts *in vitro* [86,116]. A small number of VIR proteins have been shown to be immunogenic and antibody responses to 2 VIR antigens were associated with protection from low birthweight in *P. vivax-*infected pregnant women [117]. It remains open to speculation as to whether VIR antigens contribute to immune evasion as with VSAs in *P. falciparum* and whether other VIR or non-VIR proteins are involved in cytoadherence of *P. vivax.*

### PfEMP1 types associated with disease outcomes

Several studies have found associations between expression of specific PfEMP1 subclasses, domains or combinations of domains (domain cassettes or DCs) and disease outcomes, supporting the idea that there are virulent *var* genes/PfEMP1 types. *Var* genes have been classified into groups A, B, C, and E and two intermediate groups B/A and B/C, based on their chromosomal location and type of upstream promotor sequence (UPS A, B, C, or E) [118]. Interestingly, parasites infecting cerebral and non-cerebral severe malaria patients often have upregulated expression of Group A and B/A *var* genes compared to uncomplicated malaria patients [119–122]. Additionally, PfEMP1 with adhesion phenotypes that are associated with severe or cerebral malaria are typically encoded by group A or B/A *var* genes, rather than group B or C [123,124]. Expression of group B PfEMP1 has also been associated with clinical or severe malaria in some studies [120,125] but not in others [126], as has expression of Group C var genes [119].

Individual *var* genes are composed of arrays of Duffy binding-like domains (DBLs) and cysteine-rich interdomain regions (CIDRs), in various combinations that can be subclassified into DBL α0.1–2, β1–10, γ1–18, δ1–9, ε1–14, ζ1–6 and CIDR α1–6, β1–7, γ1–12, γ1–2 [118,127]. Domain subclasses range from 38% to 98% sequence homology between parasite genomes of laboratory adapted parasite lines [118]. Some domains have been shown to adhere to specific endothelial cell receptors and have been associated with disease presentations. Adhesion to CD36 is the most common property of PfEMP1 and occurs via the CIDRα of the head structure – specifically, CIDRα2–6 domains of Group B or C PfEMP1 [128,129] that are present in 70% of *var* genes [130]. Adhesion to CD36 has been frequently associated with parasites causing uncomplicated, rather than severe malaria [130–133]. CD36 is not expressed by brain endothelial cells [134] and IE binding to human brain endothelial cells *in vitro* is independent of CD36 [135], but platelets which express CD36 can act as a bridge between IEs and brain endothelial cells [136]. Thus, the role of CD36 adhesion in cerebral malaria remains unclear. The major variants of PfEMP1 that have been associated with specific malaria syndromes are those that bind EPCR (involved in severe malaria); variants that bind both ICAM-1 and EPCR (involved in CM); a unique variant, VAR2CSA, that binds CSA (involved in placental malaria); and variants that bind uninfected RBCs (involved in rosetting), as will be discussed below.

### EPCR binding PfEMP1 associated with severe malaria

Group A PfEMP1s with head structures containing a CIDRα1.1 or 1.4–1.8 domain bind to EPCR and this includes conserved domain cassette 13 (DC13, group A) and domain cassette 8 (DC8, group B/A) [137]. EPCR is expressed on multiple endothelial cell types, and IEs containing parasites that express DC8 and DC13 are able to bind to brain, lung, heart, dermis and bone marrow endothelial cells [138]. Transcription of DC8 and DC13 has been shown to be upregulated in parasites causing cerebral malaria [121,123] and various individual group A and B CIDRα1 domains predicted to bind EPCR have also been associated with cerebral malaria [132,139–141]. Evidence is inconsistent as to whether particular CIDRα subclasses may be contributing to a specific malaria pathology, such as brain swelling [140], retinopathy positive cerebral malaria [140,142], severe malarial anaemia [123,142], and respiratory distress [143]. Further studies are needed to understand the mechanisms by which EPCR binding IE directly or indirectly contribute to severe or cerebral malaria.

### Dual ICAM-1-EPCR binding PfEMP1 associated with cerebral malaria

Post-mortem immunohistochemical studies of cerebral malaria patients first showed ICAM-1 mediated adhesion of IEs to cerebral blood vessels [134]. ICAM-1 is upregulated on brain vascular endothelial cells by inflammatory cytokines such as TNF [135] and is widely expressed in the brain, like EPCR, but unlike CD36 or E-Selectin [134]. ICAM-1 binding is mediated by DBLβ domains, including the DBLβ3 domain of group A DC4 [124,144]. A short sequence of amino acids known as the Lennartz ICAM-1 binding motif, can be used to predict ICAM-1 binding for all group A and some group B/A DBLβ3 domains [124,145]. Group A, ICAM-1 binders that contain the Lennartz ICAM-1 binding motif also have an upstream CIDRα1 domain that can bind to EPCR. Group B or C ICAM-1 binding IE (that do not have the motif) often bind to both CD36 and ICAM-1 [124].

Expression of *var* genes containing the ICAM-1-binding motif, that are predicted to induce binding to ICAM-1 and EPCR, is thought to be involved in the pathogenesis of cerebral malaria [33]. Transcripts of *var* genes containing the ICAM-1-binding motif are upregulated in parasites from patients with cerebral malaria compared to severe anaemia and uncomplicated malaria [124,140] and in severe malaria compared to uncomplicated malaria [146]. Dual ICAM-1-EPCR binding IE were more common in patients with cerebral malaria than in patients with uncomplicated malaria, whereas CD36 binders were more frequently found in patients with uncomplicated malaria [132]. In a study of parasite transcripts from 45 children, expression of *var* genes predicted to bind to EPCR but not ICAM-1 was associated with severe malaria but not with cerebral malaria or severe malaria anaemia alone, whereas transcription of *var* genes encoding both ICAM-1 and EPCR binding was associated specifically with cerebral malaria [124]. In a recent study, CIDRα-DBLβ1/3 domains predicted to bind ICAM1 and EPCR were similarly expressed in isolates causing uncomplicated and cerebral malaria [139].

### CSA binding PfEMP1 VAR2CSA is associated with placental malaria

Perhaps the most well-characterized *var* gene is *var2csa. Var2csa* is highly conserved – the 6 commonly-identified DBL domains and interdomain regions have 58–90% sequence homology across 7 different parasite genomes – and has a unique promotor sequence, UPS E [118]. *Var2csa* is the dominant *var* gene transcribed in parasites infecting the placenta [114,147,148]. VAR2CSA binds to chondroitin sulphate A, a glycosaminoglycan expressed on placental syncytiotrophoblasts [114], via the DBL2× domain and adjacent interdomain regions [149], to mediate placental sequestration of IE. Placental malaria infection is associated with poor birth outcomes [1].

## Protective immunity against severe malaria

Sterile immunity (i.e. absence of infection) is not acquired with natural exposure to malaria, however protective immunity against high-density parasitaemia and clinical disease is acquired with repeated exposure and is lost if exposure is interrupted (reviewed in [150]). Exposure-dependent acquisition of immunity likely explains why severe and clinical malaria is most prevalent in children under 5, although maturation of the immune system with age also likely contributes to protection [151]. Immunity to severe, non-cerebral malaria is acquired after 1 or 2 childhood infections in high transmission settings [152], whereas in low and moderate transmission settings the acquisition of immunity may be slower and result in a higher likelihood of severe disease [150]. This raises concerns about the impacts of interventions that reduce the incidence of malaria partially but not dramatically [153] and highlights the importance of understanding markers and mechanisms of naturally acquired immunity.

A pioneering study in the 1960s showed that transfer of immunoglobulin G (IgG) from serum of immune adults to non-immune children with malaria could dramatically reduce blood-stage parasite burden and fever, whereas transfer of non-immune serum and immunoglobulin-depleted serum had a far lesser effect [154]. This study suggested that malaria antigen-specific antibodies are important mediators of parasite clearance and protection from blood-stage malaria infection, and initiated the search for targets of IgG that may be valuable vaccine candidates or biomarkers of disease. Protein microarrays have allowed for large scale screening of immunogenicity to hundreds of antigens, including whole protein families [155] or dominant antigens that are associated with severe malaria. There are now multiple known targets of antibodies at different stages of the parasite life cycle, however a relatively small number of antigens have been associated with protective immunity and fewer have been developed into vaccines. The following sections focus on the contribution of antibody mediated immunity to protection from malaria at the various parasite lifecycle stages.

### Immunity to mosquito/sexual stage antigens

Little is known about the contribution of the immune system to reduced levels of circulating or sequestering gametocyte IEs. Stage 1 gametocyte IEs are phagocytosed by monocytes and macrophages *in vitro*, likely via CD36 recognition of trypsin-sensitive surface antigens [156]. Naturally acquired antibodies to the surface of gametocyte IEs have been reported [157] however antibody levels are dramatically lower than those to PfEMP1 on IEs [158] and the functions of these antibodies are largely unknown. Antibodies to gametocyte surface antigens, such as Pfs230 and Pfs48/45, are more prominent than responses to the gametocyte IEs surface [158] and are associated with reduced infectivity of mosquitoes fed on *P. falciparum* gametocytes grown *in vitro* [159] or in individuals [160]. Early studies reported gametocyte-specific antibodies that reduce mosquito infectivity with *P. vivax* [161] although the target antigens are not well characterized. Antibodies targeting gametocyte stage antigens are taken up with gametocytes by mosquitoes during a blood feed, and they can inhibit mosquito stage development of the parasite’s lifecycle [162]. Gametocyte, zygote, and oocyst antigens present attractive opportunities to develop transmission blocking vaccines against antigens such as Pfs25/Pvs25 and Pfs28 that are not exposed to the human immune system but may be targets of vaccine induced immunity [163].

### Immunity to pre-erythrocytic stage antigens

CD8+ T cells are important in defence against pre-erythrocytic stage parasites [164]. Individuals develop CD8+ T cells specific for the circumsporozoite protein (CSP) in malaria endemic areas and following vaccination [165], and in vivo imaging of mouse livers has shown CD8+ T cells primed for CSP can locate and destroy *Plasmodium* in infected hepatocytes [166].

CSP is the major antigen coating sporozoites prior to invasion of hepatocytes. Antibodies to *P. falciparum* CSP are naturally acquired with age [167], albeit slowly, and can promote opsonic phagocytosis [167], inhibit hepatocyte invasion and inhibit sporozoite motility [168,169]. Antibodies to *P. vivax* CSP are acquired following controlled human malaria infection and are sustained in the absence of re-infection [170]. Sterile protection does not occur naturally and naturally acquired antibodies to *P. falciparum* or *P. vivax* CSP have been associated with protection from clinical malaria in some studies but not others [168,171–173]. Despite this, the first WHO endorsed vaccine for malaria, RTS,S/AS01, is a virus-like particle made up of a recombinant fragment of CSP coupled with hepatitis-B surface antigen. It has been successfully implemented in Ghana, Kenya and Malawi [174], and endorsed for wider use. Antibodies to CSP are induced by vaccination and can promote phagocytosis and fix complement [175]. RTS,S/AS01 was estimated to provide 36% protective efficacy for clinical malaria over 4 years in a phase 3 clinical trial [176] but may be improved by administering a fractional, rather than full, booster dose at 12 months [177]. The R21 vaccine, a variation to RTS,S (that displays a higher ratio of CSP to hepatitis B surface antigen), showed more than 70% protective efficacy against uncomplicated malaria in a phase 2b clinical trial [178]. In an area of highly seasonal malaria, annual vaccination with RTS,S prior to the malaria season combined with seasonal chemoprophylaxis had a protective efficacy of 63% against clinical malaria, 70% against hospitalization with SM and 73% against death, when compared to seasonal chemoprophylaxis alone [179]. The ongoing development of RTS,S/AS01, which initially showed modest protective efficacy, has arguably set precedence for continued development of vaccines that show low efficacy in phase 1 and 2 clinical trials.

Additionally, live-attenuated sporozoites or live sporozoites administered simultaneously with chemoprophylaxis can induce sterile protection from infection against homologous controlled human malaria infection (CHMI) challenge [180]. The vaccines showed limited efficacy against heterologous parasite challenge [181] but have been shown to invoke sterile protection when co-administered with prophylactic drugs [182]. Efficacy was significantly lower in individuals from malaria endemic regions compared to US malaria naïve individuals [180] however altered dosing regimens may overcome these differences [183]. In a recent phase 2 clinical trial in children aged 5–12 months, the attenuated sporozoite vaccine gave no significant protection from infection, possibly due to poor T cell immunity, suggesting these vaccines may not be efficacious in this age group [184].

### Immunity to blood-stage antigens – merozoites

During the blood stage of infection, monocytes, neutrophils and dendritic cells recognize excess GPI on the merozoite via Toll like receptors (TLRs, a type of pattern recognition receptor), as well as haemozoin and parasite DNA released from the ruptured RBC. This initiates cell killing mechanisms such as phagocytosis and the production of proinflammatory cytokines that activate NK cells and T cells [185]. The innate immune system is enhanced by circulating serum factors including complement components and mannose binding lectin, that lead to pore insertion in the pathogen cell membrane and osmotic lysis of merozoites, but not IEs [186].

Antibodies to *P. falciparum* merozoites function to block erythrocyte invasion ligands [187,188] as well as activate the complement cascade and lead to phagocytosis [189–191]. Studies have primarily focused on antibody responses to merozoite proteins involved in invasion or in high abundance on the merozoite surface, including merozoite surface proteins (MSPs), erythrocyte binding proteins (EBAs), reticulocyte binding protein homologues (RHs) and apical membrane antigen-1 (AMA1) [192], although multiple other merozoite antigens exist [193,194]. Antibody responses to several merozoite antigens including *P. falciparum* glutamate-rich proteins (GLURPs), MSP1, MSP2, MSP3, MSP7, RH5, RIPR, and EBA175 have been associated with protection from experiencing an episode of symptomatic infection or protection from symptomatic compared to asymptomatic infection [195–200]. Several vaccines containing merozoite antigens have reached phase 2 clinical trials and are under continued development, including AMA1 [201], Rh5 [202], and combined GLURP and MSP3 [203]. Of note, recently, Rh5.1 vaccination was found to significantly reduce parasite growth rate following CHMI [202].

However, other studies have reported a lack of association between antibody responses to merozoite antigens and protective immunity, including in regards to MSP1 or AMA1 [204], or have found that protective associations are limited to older children [205]. Antibody acquisition is antigen specific, with antibodies to some antigens acquired at a young age and to others later in life or after exposure to high parasitaemia [206]. It has been suggested that antibodies to merozoite antigens may be more useful as markers of exposure [207–209]. Similarly, antibodies against *P. vivax* merozoite antigens can be used to predict recent infection [210,211]. However, further research is required to understand which antigens are most appropriate to monitor for exposure in varying populations, such as those in high transmission rather than low transmission settings [212].

### Immunity to infected erythrocyte surface antigens

Innate immune cell scavenger receptors recognize surface markers on the IE membrane, such as lipid phosphatidylserines that are usually confined to the inner-erythrocyte membrane [213], increased Band 3 protein [214] and PfEMP1 [215], and IE recognition can initiate phagocytosis of the IE and production of proinflammatory cytokines and activation of NK cells and T cells [216]. Interestingly, PfEMP1 variants have been shown to directly contribute to NK cell activation and cytokine secretion [217,218] and other PfEMP1 variants may have an inhibitory effect [219], as do some RIFIN variants [220]. It is therefore possible that antibodies targeting specific PfEMP1 variants may either suppress or enhance antibody independent NK cell activation during the blood stage of infection.

PfEMP1 is the dominant IE VSA targeted by antibodies, since parasites that do not express PfEMP1 have markedly lower antibody recognition by immune sera [221]. Several studies have found an association between antibodies to certain variants of PfEMP1 and protection from severe, cerebral or uncomplicated malaria, and will be summarized below. Almost all RIFINs, STEVORs and SURFINs are also immunogenic [222], although less studied. Antibodies to specific variants have been associated with a reduced prospective risk of developing febrile malaria [222] and a lack of antibodies to specific RIFINS and STEVORs was identified in Malian children with severe malaria compared to uncomplicated malaria [223]. Other more conserved antigens have been identified on the IE surface [224] although less is known about their immunogenicity.

Antibodies targeting trophozoite and schizont stage IEs have multiple functions, including inhibiting rosetting [89]; blocking adhesion to endothelial/epithelial cell receptors [119]; opsonising IE for phagocytosis by monocytes [205,225]; or initiating NK cell mediated lysis [217,226]. The contribution of each of these antibody mediated mechanisms to reduce blood-stage infection and protect from severe malaria requires further study. Opsonic phagocytosis of IE displaying PfEMP1 associated with severe malaria has been associated with protection from severe malaria [227] and opsonic phagocytosis of IE expressing VAR2CSA with protection from malaria in pregnancy [228] however it is not clear if some variants are better at inducing cytophilic, opsonising antibodies than others. Additionally, antibodies targeting late-trophozoite stage antigen glutamic-acid-rich protein (GARP) initiate programmed cell death *in vitro* and a lack of anti-GARP antibodies has been associated with risk of severe malaria in one study [229]. In the late schizont stages, IEs become permeable and antibodies can access intracellular antigens [230]. Antibodies that bind to intracellular Schizont Egress Antigen-1 (SEA-1) have been associated with protection from severe malaria [231,232] although the mechanism of protection are unclear [233]. Unlike merozoites, it is thought that IEs have mechanisms to evade complement fixation and lysis, possibly by upregulating complement regulatory proteins such as CD59 [186], or the orientation of PfEMP1 on knobs may prevent antibody hexamerization required for complement fixation [234]. In a recent study, complement component-1 (C1s) cleaved PfEMP1 at semi-conserved interdomain sites, found in approximately 80% of PfEMP1, which suggests that this may be a mechanism to reduce endothelial cell binding when the host is in a hyperinflammatory state and at risk of dying, or to escape opsonic phagocytosis [235].

### Antibodies targeting specific PfEMP1 domains and associations with protection from severe malaria

Due to the key pathogenic role of PfEMP1 in mediating sequestration and rosetting during blood-stage *P. falciparum* infection and the clear associated of particular *var* genes or PfEMP1 receptor binding phenotypes with severe cerebral and placental malaria, several studies have hypothesized that antibodies targeting virulent PfEMP1 types may protect from severe malaria. Antibodies to severe forms of malaria are acquired early in life in high transmission settings and severe infections are most common in children under 5 [236]. In line with a role of group A or B/A PfEMP1s in severe infections, antibodies to group A or B/A domains are more common in children under 5 and are generally acquired more rapidly than antibodies to group B, B/C, or C domains [126,237], although this pattern was only found for DBL and not CIDR domains in one study [238]. Preferential acquisition of antibodies targeting group A PfEMP1 in children may be due to the greater sequence conservation of group A *var* genes than other *var* genes or may reflect preferential expression of group A *var* genes in the naïve human host compared to hosts with pre-existing immunity, as shown in a CHMI study [239].

### Antibody responses to VAR2CSA and malaria in pregnancy

In high transmission settings, women in their first pregnancy and men have low levels of VAR2CSA-specific antibodies and women acquire VAR2CSA-specific antibodies in a gravidity-dependent manner [240]. Although antibodies to VAR2CSA block adhesion of IEs to CSA [240], a systematic review found that levels of antibodies to VAR2CSA and VAR2CSA expressing IEs measured at delivery are markers of placental infection, rather than markers of protection [241]. Rather than total levels of antibodies, a combination of IgG3 and functional antibody responses, including binding inhibition and phagocytosis, could collectively predict protection from placental infection in a recent study [225]. The glycosylation state of antibodies to VAR2CSA may also be important for protection. Naturally acquired antibody to PfEMP1 is often afucosylated, which increases its ability to interact with FcγRIIIa, expressed on NK cells and others [242].

The relationship between antibodies to pregnancy-specific IE and low birthweight is complex. Antibodies to VAR2CSA-expressing IE have been associated with reduced risk of low birthweight in some studies [243–246], but antibodies to individual VAR2CSA domains were not associated with birthweight or gestational age in another study [247]. In a recent meta-analysis, there were no significant associations between IgG responses to VAR2CSA domains and low birthweight [241]. Two vaccines have been developed based on the minimum CSA binding domain of VAR2CSA, PRIMVAC [248] and PAMVAC [249]. Both vaccines were well tolerated and immunogenic in Phase 1a clinical trials, but gave limited antibody recognition of heterologous IE [248]. PRIMVAC did not elicit antibodies that inhibit binding of IE to CSA *in vitro* [248] and it will be interesting to know whether these vaccines induce other functional antibodies. Antibodies to full- length VAR2CSA proteins show greater cross reactivity and future vaccines might need to include multiple VAR2CSA proteins, or more extensive sections of the protein [250]. While the role of VAR2CSA outside of placental malaria is still not clear, there is hope that vaccines against other PfEMP1 types will be developed to combat severe pathologies such as CM.

### Antibody responses to CD36 binding phenotype associated with UM

IE that adhere to CD36 have generally been associated with infections causing uncomplicated rather than severe malaria and antibodies targeting CD36-binding CIDRα among other domains, are predictive of a reduced prospective risk of clinical malaria and severe malaria in some studies [155,251]. Malian children with cerebral malaria lack antibodies to both CD36 and non-CD36 binding PfEMP1 fragments compared to uncomplicated malaria [223]. It seems presently unclear whether antibodies to CD36 binding PfEMP1 contribute to protection from severe malaria.

### Antibody responses to EPCR binding IE associated with SM

In line with a role of EPCR binding CIDRα1 domains in severe malaria, antibodies against these domains or downstream domains from EPCR binding PfEMP1, are significantly elevated after an episode of severe malaria [227,252] and cerebral malaria [223]. Antibodies targeting EPCR binding domains or downstream domains are associated with protection from severe malaria [227,251,253] as well as reduced prospective risk of uncomplicated malaria in one study [155] (Table 1). In contrast to these protective associations, a recent study showed that antibodies to CIDRα1.4 in young Malian children were predictive of prospective infection with higher parasite density [255]. Further studies are needed that examine functional antibodies to EPCR-binding PfEMP1s and to IEs expressing these domains. Such studies can help determine whether naturally-acquired antibody to these domains is protective, and can help identify the exact targets and features of protective antibody responses.

**Table 1.** Key PfEMP1 types associated with clinical outcomes and evidence of antibody-mediated protection from any clinical outcome. Summarizes studies included in the text only.

Adhesion/functional phenotype	Associated domains involved in adhesion	Upstream promotor Sequence (UPS) type	Clinical outcome associated with expression	Evidence of antibodies to relevant recombinant proteins being associated with protection from any clinical outcome	Evidence of antibodies to IE expressing relevant PfEMP1 being associated with protection from any clinical outcome
CD36	CIDRα2-6	B or C	Uncomplicated malaria	- reduced prospective risk of uncomplicated malaria [155] - reduced prospective risk of severe malaria [155,251] - no difference in severe and uncomplicated malaria [252].	
ICAM+CD36	DBLβ lacking Lennartz motif + CIDRα2-6	B or C	Uncomplicated malaria	- no evidence of reduced risk of uncomplicated or severe malaria (for DBLβ lacking the motif) [155,254]	
EPCR	CIDRα1.1 or 1.4–1.8	A or B	Severe malaria (multiple pathologies, including cerebral malaria)	- Protection from severe malaria compared to uncomplicated malaria (for CIDRα1 of IT4VAR19) [227]. -Protection from severe malaria compared to uncomplicated malaria (for CIDRα1.6) [253] - predictive of prospective infection with higher parasite density (for CIDRα1.4) [255] - reduced prospective risk of an episode of uncomplicated malaria [155] - reduced prospective risk of severe malaria (for DBLβ domains adjacent to EPCR binding CIDRα domains) [251]	Protection from severe malaria compared to uncomplicated malaria (for DC8 expressing parasite line, IT4VAR19) [227]
Rosetting	DBLβ1	A, B or C	Severe malaria and cerebral malaria	- Protection from severe malaria compared to uncomplicated malaria [256,257] - reduced prospective risk of severe malaria [251] - reduced prospective risk of uncomplicated malaria [237].	- Protection from cerebral malaria compared to uncomplicated malaria (for clinical isolates) [89] - Protection from severe malaria compared to uncomplicated malaria (for FCR3S1.2 parasite line) [257]
ICAM1+EPCR	DBLβ with Lennartz motif +CIDRα1.1/1.4–1.8	A	Cerebral malaria	-reduced risk of uncomplicated malaria (for DBLβ with the motif) [254] - reduced risk of uncomplicated malaria (for domains of DC4, CIDRα1.3-DBLβ3-DBLβ3) [155] - Protection from cerebral malaria compared to uncomplicated malaria overall, but not amongst individuals infected with associated domains (for Group A DBLβ3 and a CIDRα1.4-DBLβ3 couplet) [139] -no evidence of protection in severe non-cerebral malaria compared to uncomplicated malaria (DBLβ with motif) [145] -no evidence of protection from cerebral malaria compared to uncomplicated malaria (for DBLβ3 with the motif) [258] - no evidence of protection from cerebral malaria compared to uncomplicated malaria (for domains of DC13) [259]	-reduced risk of non-cerebral severe malaria (parasites expressing PF11_0521) [126] - reduced risk parasitemia, anemia and incidence of malaria fever (for CIDRα domain of dual binding PfEMP1) [260]
CSA	DBL2X and adjacent interdomain regions	E	Placental malaria	- associated with placental infection (for full length VAR2CSA and vaccine antigen) [241]. -lack of evidence of protection from low birthweight [241]. - IgG3 and functional antibody responses, associated with protection from placental infection [225].	- associated with placental infection [241]. -lack of evidence of protection from low birthweight [241]. - functional antibody responses associated with protection from placental infection [225].

### Antibody responses to dual ICAM-1 and EPCR binding IE associated with CM

Antibodies against the DBLβ3 domains with the Lennartz motif have been associated with reduced risk of uncomplicated malaria [155,254] and non-cerebral severe malaria [126], and are boosted after an episode of severe malarial anaemia [223]. In other studies, there was no difference in antibodies between uncomplicated and severe non-cerebral malaria [145], or uncomplicated and cerebral malaria [258]. Although expression of dual ICAM-1-EPCR binding PfEMP1 is associated with cerebral malaria and antibodies targeting these domains can efficiently block adhesion of IE *in vitro* [145], further studies are needed to confirm if naturally acquired antibodies to these parasites are associated with protection from cerebral malaria. In a recent study, Beninese children with uncomplicated malaria had higher antibodies to a recombinant Group A DBLβ3 and a CIDRα1.4-DBLβ3 couplet (predicted to be dual ICAM-1 and EPCR binding) than children with cerebral malaria. No difference between cerebral and uncomplicated malaria was observed in the subset of children confirmed to be infected with parasites expressing Group A DBLβ3 with the motif (presumed to be dual binders), although only a single recombinant DBLβ3 was tested [139]. Interestingly, in another small cohort, total IgG targeting full length DC13 (which is dual ICAM-1 and EPCR binding) was boosted in individuals with uncomplicated malaria from presentation to convalescence, whereas IgG1 and IgG3 to this protein were boosted in convalescence from cerebral malaria [259]. This suggests that the quality of the antibody response to dual ICAM-1 and EPCR binding PfEMP1 may be a more effective correlate of protection from cerebral malaria.

### Antibody to domains associated with rosetting

Antibodies raised against recombinant DBLβ1 domains of rosetting parasite lines are able to bind and inhibit rosette formation [256,261] and induce phagocytosis of IEs [262]. Antibodies to recombinant domains predicted to be from rosetting parasite lines are elevated in uncomplicated malaria compared to severe malaria and are associated with reduced prospective risk of severe malaria [251,257] and uncomplicated malaria [237]. Furthermore, antibodies against rosetting parasite lines were elevated in children with uncomplicated malaria compared to severe malaria and to children with cerebral malaria [89,257].

### Cytokine response

A strong cytokine response is triggered by *Plasmodium-*derived toxins that are released into circulation during cell lysis, including glycosylphosphatidylinositol (GPI), haemozoin, and cell-free DNA and RNA. Toxins are recognized by pattern recognition receptors, including TLR4 on the surface of innate immune cells, TLR7, TLR8, and TLR9 on endosomes and cytosolic cGAS and AIM2 to induce production of a type 1 interferon response and pro-inflammatory cytokines (reviewed in [263]). During the liver stage of infection, hepatocytes detect sporozoite RNA via cytosolic MDA5 to produce a Type I IFN response that primes NK cells, NKT cells and DCs and recruits CD8+ T cells, although the contribution of this response to preventing subsequent disease is unclear [263,264]. Malaria is associated with an increase in circulating pro-inflammatory cytokines, including IL-1α, IL-1β, IL-6, IL-12, IL-18, and TNF-α, as well as anti-inflammatory cytokines, particularly IL-10, and chemokines including MIP1, IL-8, CCL5, and others [185,265]. Cytokines and chemokines recruit and activate innate immune cells including neutrophils, monocytes, macrophages, dendritic cells, and NK cells for innate immune killing mechanisms, such as phagocytosis [185]. Interestingly, *P. falciparum* infection leads to epigenetic changes in monocytes that increase the level of secretion of IL-6 and TNF-α upon secondary stimulation one month after infection [266], suggesting “trained immunity” affects cytokine responses and potentially contributes to long-term protection.

Alongside the importance of type 1 interferons and pro-inflammatory cytokines/chemokines in mounting an effective immune response against liver and blood-stage malaria, several studies have associated a dysregulated pro-inflammatory cytokine response with severe malaria, cerebral malaria, and death [265,267–269]. Larger cohort studies using consistent markers are required to evaluate whether a particular cytokine combination may have prognostic value for severe clinical outcomes [265,270]. Cytokines activate human endothelial cells *in vitro* [271] and circulating cytokines have been proposed to contribute to localized brain inflammation in patients with cerebral malaria, ultimately leading to impaired blood–brain barrier integrity and brain swelling that is associated with death [33,265]. Endothelial cells (including human brain endothelial cells) also secrete cytokines when stimulated with IEs *in vitro* [272] that may perpetuate localized inflammation *in vivo*. In a study of children with cerebral malaria, there were no differences in peripheral cytokine levels of children with severe brain swelling compared to no severe brain swelling [273]. This may reflect differences in the peripheral cytokines compared to a local environment or suggest that secondary factors are involved in brain swelling [273].

Malaria also disrupts the balance of pro-inflammatory and anti-inflammatory cytokines required for a healthy pregnancy and several maternal cytokines have been associated with poor pregnancy outcomes including preterm birth, small for gestational age babies, and pregnancy loss [274–276]. Placental inflammation is strongly linked to poor pregnancy outcomes, due to impaired placental development or function (see section 9.4), and the local placental cytokine environment can differ significantly from the maternal periphery [277], adding a layer of complexity to finding prognostic markers.

## Molecular basis of severe disease phenotypes and symptoms

### Cerebral malaria

Unarousable coma from malaria is known as *cerebral malaria*. Histologically, it is characterized by the sequestration of IE in the brain, typically in the capillaries, as well as pre-capillary arterioles and post-capillary venules. In the majority of cases, sequestration is accompanied by microvasculature pathologies, particularly haemorrhages in the brain white matter, thrombosis and accumulation of monocytes [63].

Cerebral malaria in children with documented malaria infection is clinically diagnosed by the Blantyre coma score, based on motor, verbal and eye movement responses. This diagnostic method overestimates cerebral malaria cases by up to 30% in a high transmission settings and up to 54% in low or moderate transmission settings [278,279]. The retina reflects changes to the brain microvasculature and retinal examination for retinal whitening and blood vessel changes can drastically increase diagnosis sensitivity and specificity [280]. A normal retinal examination in individuals with impaired consciousness and parasitaemia should lead to consideration of other causes of coma, but may also reflect an earlier stage of cerebral malaria with retinopathy [281,282]. An incorrect clinical diagnosis of cerebral malaria may have devastating consequences, resulting in delayed treatment of alternative, life threatening conditions, and may underpin discrepancies in surveillance and epidemiological research.

Increased brain volume due to swelling in the brain white matter [283] is common to almost all children who die from cerebral malaria [284] and the final cause of death is respiratory arrest due to pressure on the brain stem [284]. In adults, swelling is more pronounced in the basal ganglia and fatal cerebral malaria is associated with severe hypoxia, rather than pressure on the brainstem [283], suggesting potentially different mechanisms of fatality in adults and children. Mortality from cerebral malaria in Africa is between 15% and 25% [285]. Amongst survivors, up to 30% experience neurological impairment, which often manifests as epilepsy, motor and language regression and greater risk of developing disruptive behavioural disorders [286–288]. The mechanisms of long-term neurological impairment are unknown. Elevated levels of tau in cerebral spinal fluid and increased transcriptions of genes linked to Alzheimer’s disease may be involved [289,290]. In a recent study, examination of the retina revealed that leaks from endothelial cell tight junctions were associated with neurological disability, whereas larger haemorrhagic breaches in the endothelium were associated with brain swelling and death [291].

**Figure 2.**
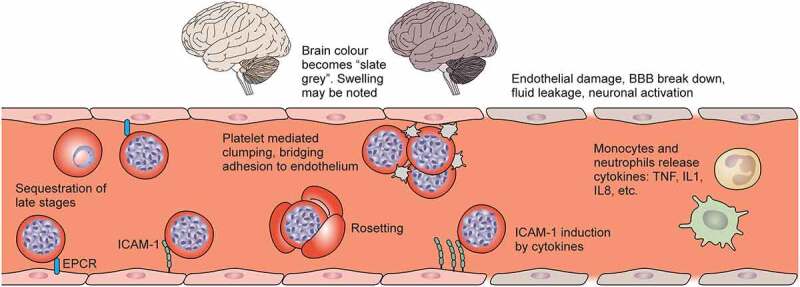
Sequestration in *Plasmodium falciparum*. Reading left to right, the brain colour changes from light tan to a “slate grey” colour in cases of fatal cerebral malaria. Mature infected erythrocytes (IEs) sequester in the deep vasculature, including cerebral vessels. Receptors on endothelial cells include endothelial protein C receptor (EPCR), ICAM-1 and heparan sulphate proteoglycans. Platelets can clump IEs, and bridge between CD36 binding IEs and endothelium. IE can activate monocytes and neutrophils to release cytokines and immune mediators, which can upregulate ICAM-1 expression, and induce damage to the blood–brain barrier. Fluid leakage through and between cerebral endothelial cells can lead to neuronal activation and may result in potentially fatal brain swelling, associated with brain discolouration.

The current understanding of the pathogenesis of cerebral malaria [33] centres around activation of the endothelial cells leading to impaired blood–brain barrier integrity and ultimately neuroinflammation, as will be summarized below (Figure 2).

Several factors have been proposed to contribute to activation and impaired integrity of brain blood vessel endothelial cells. Sequestration of IEs in the brain microvasculature is strongly associated with coma and it has been hypothesized that microvascular congestion due to sequestration of IEs, platelet mediated clumping or rosetting causes hypoperfusion and ischaemic damage to the endothelium [292]. IE, particularly in the schizont stage, can directly activate endothelial cells and trigger apoptosis [33]. EPCR binding IE compete for EPCR binding with activated protein C (APC), involved in regulation of blood coagulation, inflammation, and endothelial cell integrity and apoptosis [293]. It has been proposed that reduced APC binding results in downregulation of EPCR, which may decrease adhesion of EPCR binding IE, but also upregulation of ICAM-1 that can preserve adhesion of ICAM-1-EPCR dual binding IE as well as increase platelet activation and endothelial barrier breakdown [33]. A recent study showed that *in vitro*, human brain endothelial cells can phagocytose dual ICAM-1-EPCR binding IE in an ICAM-1 dependent manner, and parasites engulfed in brain endothelial cells were observed in *ex vivo* samples from two cerebral malaria patients [294], highlighting a novel potential pathway of endothelial cell activation or defence.

In addition to IE, several parasite factors are thought to contribute to endothelial cell activation, including haemozoin and products bound to haemozoin [295], HRP2 [296], histones [297], and kinins [298] released from IE. Local inflammatory factors have also been shown to contribute, such as matrix metalloprotein 8 released from leukocytes, reduced endothelial nitric oxide bioavailability, and pro-inflammatory cytokines [33,265], although neither inhaled nitric oxide nor anti-TNFα therapy showed any potential as adjunctive therapies [299,300]. In the *P. berghei* ANKA mouse model of experimental cerebral malaria (ECM), CD3+CD8+ T cells in the brain vasculature are considered key drivers of intense inflammation and disruption of the blood–brain barrier [301]. Recent studies suggest that similar T cells are also present in the brains of children with cerebral malaria [302], although with potentially different compartmentalization [303], therefore the role of T cell accumulation in the brain of children with cerebral malaria requires further elucidation.

In response to IEs, parasite factors and inflammatory mediators, the activated endothelium expresses local inflammatory cytokines to perpetuate inflammation and dysregulates factors that mediate endothelial cell growth and integrity, including angiopoietin 2 and vascular endothelial growth factor [33]. The activated endothelium also creates a pro-coagulant stage that perpetuates endothelial barrier breakdown [272,304]. Platelets are activated, becoming capable of killing IEs by release of Platelet Factor 4 [305], and additionally contribute to IE sequestration [136,306] and endothelial apoptosis [307].

In summary, multiple factors contribute to endothelial cell activation, breakdown, and impaired blood–brain barrier integrity, and there is strong evidence for a role of sequestration and cytoadherence, particularly of dual EPCR-ICAM-1 binding IE. Further studies are needed to understand the relative importance of these factors and the mechanistic links to clinical outcomes of cerebral malaria, including coma, severe brain swelling that leads to death, and long-term neurological sequelae.

### Severe malarial anaemia

Severe anaemia, defined as haemoglobin <7 g/dL in adults and <5 g/dL in children, is a common cause of death in Africa [1], particularly in malnourished children [308] and malaria is a significant risk factor for severe anaemia, particularly in children under five years of age [309]. According to a large household survey conducted in 16 malaria endemic African countries, 79% of children under five years of age with malaria had anaemia (haemoglobin <12 g/dL) and 8% had severe anaemia [1]. Parasitaemia in severe anaemia is generally low, and the loss of IE alone is not sufficient to account for the extremely low haemoglobin, although severe malaria anaemia is associated with parasitaemia [310]. Anaemia has been associated with destruction of large numbers of non-IEs [311] and evidence suggests this may be attributed to increased erythrocyte rigidity leading to increased splenic clearance [312].

Several studies have suggested a key role of systemic inflammation in the pathogenesis of severe malaria anaemia. The release of parasite factors during IE rupture can induce complement deposition on nearby non-IEs leading to the latter’s phagocytic clearance [313]. Additionally, complement regulatory proteins are reduced on the erythrocyte surface in severe malaria anaemia, most notably on uninfected cells, whereas IEs have higher levels of complement regulatory proteins that may be protective [314].

IEs and secreted parasite factors may contribute to severe anaemia by the disruption of erythropoietic processes. *In vitro*, IEs directly alter transcription of globin mRNA [315]. Secretion of parasite factor haemozoin may inhibit erythropoiesis directly [316] or indirectly via factors released from macrophages following phagocytosis in the bone marrow [317]. Additionally, systemic inflammatory factors [269,318] and pro-inflammatory cytokines have been associated with SMA [319,320] and may upset erythrocyte homoeostasis to reduce erythropoiesis [321].

Delayed haemolytic anaemia following artemisinin treatment was first observed following infection in non-immune travellers [322] and is thought to be due to the reduced lifespan of erythrocytes that are rapidly pitted by the spleen following artemisinin treatment [322]. A significant drop in haemoglobin following treatment has been observed in up to 5% of patients [323,324] and can be severe [325] but there is debate as to whether this is specifically associated with artemisinin treatment [323,326,327]. Other studies in Africa have found no evidence of delayed haemolysis [328] or rare incidence of post treatment anaemia [329]. These discrepancies may be partially explained by differences in exposure. Post-artemisinin anaemia appears to be more common in non-immune travellers [322] compared to individuals living in endemic settings [327] and a recent report found it was more common in low transmission settings (Asia) compared to high transmission settings (Africa) [310].

### Acute kidney injury (AKI)

The WHO defines Acute Kidney Injury (AKI, renal impairment) as impaired glomerular filtration, based on serum creatine of >3 mg/dL or blood urea >20 mM [330]. However, the Kidney Disease: Improving Global Outcomes (KDIGO) Acute Kidney Injury working group recommends using serum creatinine of <0.3 mg/dL in 28 h or urine volume below 0.5 mL/kg/h. Under the KDIGO definition, AKI is a manifestation of severe *P. falciparum* malaria in both adults [331] and children [332], including in conjunction with severe malarial anaemia and cerebral malaria [332]. AKI has also been reported in *P. vivax* [331,333], *P. knowlesi* [333] and occasionally *P. malariae* [334]. AKI is associated with an increased duration of hospitalization and mortality [332,335] and potentially an increased risk of chronic kidney disease, particularly in children with cerebral malaria.

Multiple molecular mechanisms of AKI in malaria have been proposed. Renal ischaemia can directly cause tubular damage or necrosis, and it may be brought about by excessive fluid loss or impaired glomerular blood flow due to parasite obstruction. Histologically, IEs (*P. falciparum*, *P. vivax* and *P. knowlesi*) and haemozoin have been observed in the glomerular capillaries of some individuals with AKI [333], although at autopsy there is less sequestration in the kidneys than in the brains of the same patients [336]. Parasite risk factors may include high parasite biomass [332], release of cell free haem from IE that directly damages renal tubules and initiates infiltrates of monocytes and neutrophils [336–338], and generation of auto anti-DNA antibodies [339]. Parasite sequestration, reduced renal blood flow and systemic and local inflammation can contribute to glomerular endothelial cell activation [335], which perpetuates inflammation and endothelial cell damage. In mice (*P. berghei* ANKA) treatment with Angiopoietin 2 or Angiopoietin 2 receptor prevented pro-inflammatory cytokine production and mouse AKI [340]. Understanding the mechanisms of AKI may lead to development of better therapeutics.

### Malaria in pregnancy

Malaria in pregnancy causes adverse health outcomes for both mother and baby. It is strongly associated with an increased risk of maternal anaemia and *P. falciparum* at any point in pregnancy is associated with low birthweight (<2,500 g) due to foetal growth restriction or preterm delivery [8]. Approximately 34% of pregnancies are exposed to malaria in Sub-Saharan Africa resulting in approximately 800,000 low birthweight deliveries each year [1]. *P. vivax* malaria in pregnancy is less well studied but has also been associated with preterm birth and low birthweight [341,342]. Both *P. falciparum* and *P. vivax* malaria in the first trimester are associated with increased risk of miscarriage, and malaria during pregnancy is associated with increased risk of *in utero* still birth [343,344].

*P. falciparum* placental malaria occurs when IE cytoadhere in the intervillous space of the placenta via VAR2CSA [114]. There are no reliable biomarkers of placental malaria before delivery, so it is diagnosed by histological examination of the delivered placenta. Placental malaria is characterized by the presence of IEs (active infection) or haemozoin without IEs (past infection) in the placenta intervillous space [345]. Placental malaria is associated with low birthweight, pre-term delivery and maternal anaemia [346]. Placental infiltrates of IEs are sometimes reported following *P. vivax* in pregnancy but seem to be uncommon [347,348]. However, evidence of placental damage is present in both *P. falciparum* and *P. vivax* during pregnancy, including syncytial knotting, fibrin deposits, increased placental barrier thickness, and presence of mononuclear cells [347,349–352].

There are multiple proposed mechanisms by which parasites in peripheral or placental blood may contribute to poor birth outcomes and inflammation is thought to be a major contributor. *P. falciparum* infection can lead to placental intervillous infiltrates of monocytes [353], T cells [354], and polymorphonuclear cells [355]. If intense, these cell accumulations are termed intervillositis, and monocytes and macrophages are the dominant cell types. Intervillositis is more closely associated with poor outcomes than parasites alone [353,356], particularly low birthweight [356–358]. The release of circulating and localized inflammatory cytokines and complement factors has been associated with poor pregnancy outcomes in *P. falciparum* [359,360], although other studies have found a protective association for some cytokines [361]. In *P. vivax* malaria in pregnancy, cytokines are also skewed towards a proinflammatory response although further studies are needed to determine the associations with poor pregnancy outcomes [362,363]. Placental angiogenesis and vascularization are disrupted in placental malaria [356,360], possibly due to inflammation and increased asymmetric dimethylarginine that impairs the nitric oxide synthesis pathway [364]. A clearer understanding of these mechanisms may lead to development of adjunctive therapies to reduce the impacts of malaria in pregnancy.

## Therapeutic intervention

Treatment of malaria is continuously challenged by the emergence of drug resistant parasites. Delayed parasite clearance with artemisinins, the frontline anti-malarial drugs, has emerged in South East Asia [365] and has been linked to several mutations in the propeller region of *P. falciparum* Kelch13 protein [366]. To curb the spread of these mutations, malaria is treated with artemisinin derivatives in combination with a partner drug such as amodiaquine, mefloquine, piperaquine, sulphadoxine-pyrimethamine (SP) or lumefantrine (to clear any remaining parasites), known as Artemisinin Combination Therapy (ACT) [4]. ACT partner drug resistance to SP, piperaquine, lumefantrine and mefloquine has been identified [367–370] and likely contributes to delayed parasite clearance following ACT in South East Asia. In Africa, ACT remains highly effective for treatment of uncomplicated malaria [371–374] and Kelch13 mutations are rare [375]. However, genetic diversification of Kelch13 has been detected in Rwanda [376] and high survival of rings and mutations associated with partner drug resistance have been reported in Uganda [377]. Reduced efficacy of artemisinin would be particularly devastating in Africa due to high rates of severe malaria, which is treated with rectal or intravenous artesunate, to rapidly kill ring-stage parasites, followed by full course of ACT [4]; therefore, continued active surveillance of mutations associated with reduced artemisinin efficacy and partner drug resistance is necessary. Local emergence of drug resistance is likely driven by drug pressure (overuse) or incomplete dosing regimes (underuse), and resistance is more likely to spread in low transmission settings [378]. In Africa and South East Asia, substandard antimalarials [379] and subtherapeutic dosing have been shown to promote growth of drug resistant parasites *in vitro* [380].

Cerebral malaria is treated with parenteral artesunate [1] and by management of associated morbidities, such as hypoglycaemia and convulsions, and few adjunctive therapies exist. Fluid bolus resuscitation was shown to increase mortality compared to use of maintenance fluids alone [381]. Similarly, routine treatment of seizures in children with cerebral malaria with the anticonvulsant drug, phenobarbitone, was associated with increased mortality in a clinical trial meta-analysis [382], although a recent small study suggested levetiracetam may be a safer alternative [383], and management of brain swelling with mannitol was associated with increased length of coma [384]. A deeper understanding of the molecular mechanisms of cerebral malaria may lead to more effective adjunctive therapies.

In areas where it is chloroquine sensitive, *P. vivax* can be treated with chloroquine or an ACT. Where chloroquine resistance occurs, an ACT is recommended [385]. In both cases, follow-up treatment with primaquine is necessary to eliminate *P. vivax* and *P. ovale* hypnozoites and prevent relapse [4]. Single dose primaquine is recommended following *P. falciparum* to reduce gametocyte burden and transmission [386]. Individuals with Glucose 6 Phosphate Dehydrogenase deficiency (G6PDd) are susceptible to haemolytic anaemia when treated with primaquine but haemolysis is stopped when treatment is stopped. G6PDd is an X-linked genetic condition caused by a large variety of mutations and reaches high prevalence in malaria endemic populations as it has a protective effect against malaria [387]. The recently approved single-dose alternative to primaquine, tafenoquine [388], also induces G6PDd dependent haemolysis but, unlike primaquine, only requires a single dose, and thus treatment cannot be stopped to limit haemolysis. Pretreatment G6PD screening is recommended but is not widely available. Current RDTs detecting G6PDd may not detect heterozygous females who may also be vulnerable to haemolysis [389,390] and such tests are not always widely available [391].

## Current and future research, barriers to effective treatment, and models of disease

**Figure 3.**
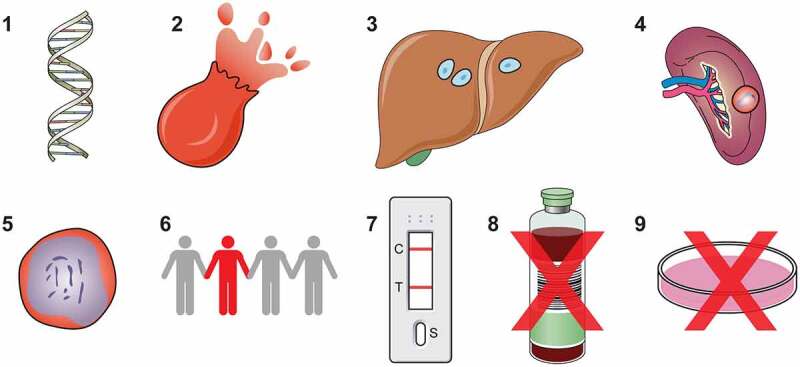
**Challenges in *Plasmodium vivax.*** *Vivax* malaria presents a unique set of challenges for elimination. 1. Genetic mutations: CYP2D6 polymorphisms decrease effectiveness of radical cure, G6PD deficiency predisposes to 2. Drug-induced haemolysis. 3. Hypnozoite reactivation in the liver causes most new episodes of *P. vivax* malaria. 4. Reservoirs of hidden infection include the spleen (illustrated) and bone marrow. 5. *P. vivax* forms gametocytes early in infection, increasing transmissibility. 6. Asymptomatic carriers of *vivax* whose infection may be submicroscopic (figure in red) are common. 7. Low-density infections may not be detected by RDTs. 8. There are no imminent prospects for a *P. vivax* vaccine. 9. Robust in vitro culture systems are yet to be developed.

We face ongoing challenges to the improved control, elimination and eventual eradication of malaria. Improved diagnostics are needed, both to circumvent the problem of HRP2 mutant parasites that evade commonly used *P. falciparum* RDTs, and to improve detection of low-density infections. In pre-elimination settings, serology holds promise, especially for detection of recent exposures to *P. vivax*. Better, more widely available point of care G6PD testing is critical to the widespread implementation of primaquine or tafenoquine to eradicate *P. vivax* hypnozoites (Figure 3).

Extending the implementation of proven effective preventives and improving vaccine efficacy are critical. The roll out of RTS,S vaccine to small children in Africa can potentially prevent thousands of deaths each year. If the efficacy of the R21 vaccine is confirmed in definitive trials, this could be a step-change in malaria prevention. Combining sporozoite proteins with gametocyte antigens has the potential to decrease infection and transmission and make inroads into the otherwise unchanging burden of malaria deaths. Similar vaccines are urgently needed for other species, most notably *P. vivax*.

At the same time, more widespread implementation of seasonal malaria chemoprevention (possibly combined with seasonal vaccination) could protect up to three times as many children from severe malaria and risk of dying each year. Increasing coverage with intermittent preventive treatment in pregnancy and determining the most effective drug combination will decrease the burden of placental malaria and associated low birthweight and neonatal and infant mortality.

New drugs and smarter ways of administering existing drugs will be key to combating the rise of drug resistance. ACT with 3 drugs offers hope in the short term, while new classes of agents are developed. Controlled human malaria infections are a powerful tool to speed up the development of new drugs, vaccines and alternative therapies such as monoclonal antibodies, which could provide sustained protection against infection [392].

Models of sequestration using organoids [294] or engineered microcapillaries will provide new insights into the mechanisms underlying sequestration [393] and may reveal novel targets for intervention to block sequestration or to prevent severe disease. These may include members of the PfEMP1 family of proteins if these prove functionally conserved and are confirmed to be major targets of anti-disease immunity. VAR2CSA, the placental malaria associated PfEMP1, is a first example. The murine experimental malaria model lacks PfEMP1, but can give insights into pathogenic processes at the cellular and molecular level. The *Aotus nancymaae* monkey model of placental malaria caused by *P. falciparum*, however, does recapitulate many of the features of human placental malaria [394].

A major difficulty with *P. vivax* elimination has been the lack of robust experimental challenge and in vitro culture systems. The liver humanized mouse model shows significant promise as a tool to study liver stage infection and development of hypnozoites [395], and a *P. vivax* CHMI has been developed [396]. More recently, bone marrow humanized mice that produce human CD71+ human red blood cells have been shown to maintain long-lasting *P. vivax* cultures [397]. Other challenges are summarized in a recent series of reviews [398]. Meanwhile the impact of *P. knowlesi* in risk groups in South East Asia remains a significant concern [399].

The lack of progress in reducing global malaria deaths in recent years highlights the problems we face in controlling this disease. While better deployment of existing tools can make a significant impact we still lack the powerful tools, such as highly effective vaccines, that could significantly accelerate progress towards eradication.
